# Supplementary material for: MicroRNA-27a-5p Downregulates Expression of Proinflammatory Cytokines in Lipopolysaccharide-Stimulated Human Dental Pulp Cells via the NF-κB Signaling Pathway
Source: Int J Mol Sci. 2024 Sep 7;25(17):9694. doi: 10.3390/ijms25179694 (PMC11395329; doi:10.3390/ijms25179694)
Supplement: Supplementary file 1 [file ijms-25-09694-s001.zip › ijms-3151988-supplementary.pdf]

Table S1. GeneChip miRNA 4.0 Array Data

miRNA expression profile of the hDPCs (P5, 2×10^5 cells) stimulated with (n=3) or without (control, n=3) LPS (*E. Coli*O111:B4, 100ng/ml) for 2 h was analyzed by the GeneChip miRNA 4.0 Array, and the miRNAs whose gene expression was upregulated by 1.5-fold or more upon LPS stimulation are listed.

	Expression value {LPS}	Expression value {control}	Ratio
hsa-miR-4324	45.00	11.81	3.81
hsa-miR-4304	32.19	8.91	3.61
hsa-miR-27a-5p	43.59	13.88	3.14
hsa-miR-16-1-3p	4.31	1.42	3.04
hsa-miR-338-5p	3.93	1.51	2.60
hsa-miR-642b-3p	11.80	4.62	2.56
hsa-miR-668-5p	5.76	2.34	2.45
hsa-miR-3124-5p	19.54	8.57	2.28
hsa-miR-4306	5.46	2.41	2.27
hsa-miR-202-3p	5.82	2.59	2.25
hsa-miR-204-3p	9.12	4.13	2.21
hsa-miR-378a-3p	23.60	11.84	1.99
hsa-miR-8085	3.97	2.04	1.94
hsa-miR-103a-2-5p	2.50	1.30	1.92
hsa-miR-23a-5p	146.64	76.69	1.91
hsa-miR-6781-5p	5.22	2.74	1.91
hsa-miR-6829-5p	3.90	2.09	1.87
hsa-miR-1281	8.20	4.54	1.81
hsa-miR-619-5p	19.46	10.84	1.79
hsa-miR-6836-5p	5.16	2.91	1.77
hsa-miR-3934-5p	2.51	1.45	1.73
hsa-miR-200a-5p	2.17	1.26	1.72
hsa-miR-6768-5p	12.17	7.09	1.72
hsa-miR-4470	2.35	1.37	1.71
hsa-miR-17-3p	2.59	1.53	1.69
hsa-miR-6748-5p	2.78	1.65	1.69
hsa-miR-30e-3p	3.21	1.94	1.66
hsa-miR-195-3p	2.82	1.71	1.65
hsa-miR-542-3p	3.55	2.15	1.65
hsa-miR-26b-3p	2.66	1.63	1.63
hsa-miR-6891-5p	23.29	14.45	1.61
hsa-miR-4791	3.68	2.37	1.55
hsa-miR-6876-5p	1.92	1.25	1.54
hsa-miR-381-3p	1.94	1.27	1.52
hsa-miR-187-5p	3.03	1.99	1.52
hsa-miR-3186-3p	1.47	0.97	1.52
